# Cholesterol‐Amino‐Phosphate (CAP) Derived Lipid Nanoparticles for Delivery of Self‐Amplifying RNA and Restoration of Spermatogenesis in Infertile Mice

**DOI:** 10.1002/advs.202300188

**Published:** 2023-02-07

**Authors:** Shi Du, Wenqing Li, Yuebao Zhang, Yonger Xue, Xucheng Hou, Jingyue Yan, Jeffrey Cheng, Binbin Deng, David W. McComb, Jennifer Lin, Hong Zeng, Xiaolin Cheng, Darrell J. Irvine, Ron Weiss, Yizhou Dong

**Affiliations:** ^1^ Division of Pharmaceutics & Pharmacology College of Pharmacy The Ohio State University Columbus OH 43210 USA; ^2^ Center for Electron Microscopy and Analysis The Ohio State University Columbus OH 43212 USA; ^3^ Department of Materials Science and Engineering The Ohio State University Columbus OH 43210 USA; ^4^ Transgenic Knockout and Tumor Model Center Stanford University School of Medicine Stanford CA 94305 USA; ^5^ Division of Medicinal Chemistry and Pharmacognosy College of Pharmacy The Ohio State University Columbus OH 43210 USA; ^6^ Department of Biological Engineering Massachusetts Institute of Technology Cambridge MA 02139 USA; ^7^ Koch Institute for Integrative Cancer Research Massachusetts Institute of Technology Cambridge MA 02139 USA; ^8^ Department of Materials Science and Engineering Massachusetts Institute of Technology Cambridge MA 02139 USA; ^9^ Ragon Institute of Massachusetts General Hospital Massachusetts Institute of Technology and Harvard University Cambridge MA 02139 USA; ^10^ Howard Hughes Medical Institute Chevy Chase MD 20815 USA; ^11^ Department of Biomedical Engineering Center for Clinical and Translational Science Comprehensive Cancer Center Dorothy M. Davis Heart & Lung Research Institute Department of Radiation Oncology Center for Cancer Engineering Center for Cancer Metabolism Pelotonia Institute for Immune‐Oncology The Ohio State University Columbus OH 43210 USA; ^12^ Icahn Genomics Institute Precision Immunology Institute Department of Oncological Sciences Tisch Cancer Institute, Friedman Brain Institute Icahn School of Medicine at Mount Sinai New York NY 10029 USA; ^13^ Synthetic Biology Center Massachusetts Institute of Technology Cambridge MA 02139 USA; ^14^ Department of Electrical Engineering and Computer Science Massachusetts Institute of Technology Cambridge MA 02139 USA

**Keywords:** lipid nanoparticles, male infertility, mRNA therapy, self‐amplifying RNA, spermatocytes delivery

## Abstract

Male infertility caused by genetic mutations is an important type of infertility. Currently, there is no reliable method in the clinic to address this medical need. The emergence of mRNA therapy provides a possible strategy for restoring mutant genes in the reproductive system. However, effective delivery of mRNA to spermatocytes remains a formidable challenge. Here a series of cholesterol‐amino‐phosphate (CAP) lipids are reported by integrating three bioactive moieties into a geometric structure, which is favorable for mRNA delivery. The results demonstrate that CAP‐derived lipid nanoparticles (CAP LNPs) can deliver RNA including traditional mRNA and self‐amplifying RNA (saRNA) encoding DNA Meiotic Recombinase 1 (Dmc1) protein in spermatocytes and treat male infertility caused by the Dmc1 gene mutation. Notably, the delivery efficiency of CAP LNPs is significantly higher than that of the MC3 and ALC‐0315 LNPs, which is consistent with the design of CAP molecules. More importantly, a single injection of CAP LNPs–saRNA can produce Dmc1 protein for an extended period, which restores the spermatogenesis in the Dmc1 gene knockout mouse model. Overall, this study proves the concept of LNPs for the delivery of mRNA to spermatocytes, which provides a unique method to probe male infertility caused by the genetic mutation.

## Graphical abstract

Cholesterol‐amino‐phosphate lipid nanoparticles (CAP LNPs) are developed to deliver mRNA and self‐amplifying RNA (saRNA) encoding Dmc1 protein to spermatocytes of Dmc1^−/−^ infertile mice. After microinjection to seminiferous tubules, CAP LNPs‐saRNA induces long‐term expression of Dmc1 protein in spermatocytes. The sufficient supplementation of Dmc1 protein rescues the chromosomes recombination, thereby recovering the meiosis and spermatogenesis.

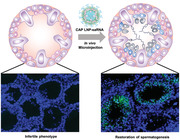

## 1 Introduction

Male infertility is an important reproductive disease, affecting at least 7% of men globally.^[^ ^1^ ^]^ Currently, certain cases of male infertility can be treated with assisted reproductive techniques (ART).^[^ ^1a^ ^]^ However, ART cannot solve the issues of male infertility caused by genetic mutations.^[^ ^2^ ^]^ Common genetic mutations that cause male infertility in humans include synaptonemal complex protein 3 (Sycp3) gene and testis‐expressed gene 11 (TEX11) mutations.^[^ ^3^ ^]^ These genetic mutations are usually associated with abnormal protein function in spermatocytes, meiotic arrest, and severe azoospermia.^[^ ^4^ ^]^ Unfortunately, there is no effective method in the clinic to restore genetic function and fertility in humans.

To address the important medical demand of male infertility, one of the challenges is to deliver therapeutic cargos into the diseased cell population such as spermatocytes. Ionizable lipid‐derived nanoparticles (LNPs) are one of the most widely used nanomaterials for mRNA delivery owing to their low immunogenicity and favorable delivery efficiency.^[^ ^5^ ^]^ In 2020, two LNP‐messenger RNA (mRNA) vaccines have been approved for emergency use against severe acute respiratory syndrome coronavirus 2 (SARS‐CoV‐2).^[^ ^6^ ^]^ Meanwhile, researchers have investigated LNP‐mRNA formulations for a variety of therapeutic applications, such as genetic engineering and protein replacement therapy.^[^ ^7^ ^]^ Although LNPs have served as an important delivery system in various tissues including liver, lung, spleen, and tumor,^[^ ^8^ ^]^ effective delivery in testis especially spermatocytes remains a formidable challenge and an underexplored field for mRNA delivery.

To facilitate the delivery of mRNA to spermatocytes, some bioactive molecules attracted our attention due to their critical roles in biological membranes and the male reproductive system. For example, cholesterol is an essential structural component of mammalian cell membranes, which maintain the integrity and permeability of membranes. Prior studies have also reported elevated cholesterol levels during spermatogenesis, indicating a close relationship between cholesterol metabolism and fertility.^[^ ^9^ ^]^ Additionally, the phosphate group is a polar part of the phospholipid which is a key component of biological membranes and participates in cell transport pathways. Lastly, the amino groups can be ionized to become positively charged, which can interact with negatively charged mRNA. In addition to the biological activity, the geometry of the ionizable lipids, which can be described by packing parameter (*P* value), can greatly affect the delivery efficiency of the LNPs.^[^ ^10^ ^]^ Typically, lipids with a large *P* value are favorable to form an inverted conical shape, which facilitates the inverted hexagonal (H_II_ phase) transformation of the endosome membrane and the endosomal escape of payloads.^[^ ^10^ ^]^

**Figure 1:**
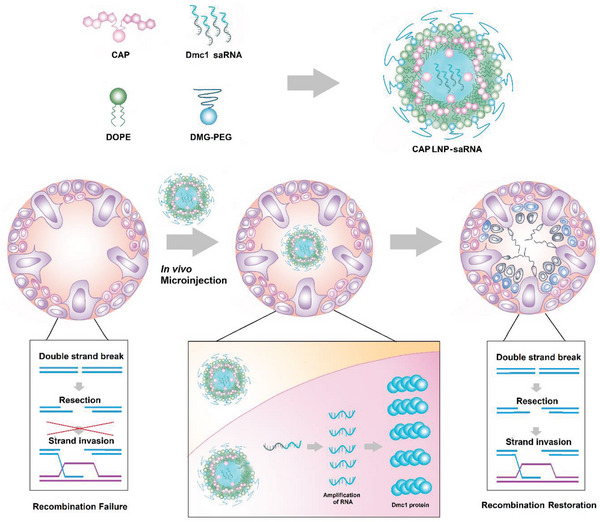
Schematic illustration of delivery of mRNA encoding Dmc1 using CAP LNPs. CAP LNPs are formulated with CAP, DOPE, DMG‐PEG, and mRNA encoding Dmc1. After microinjection to seminiferous tubules, CAP LNPs deliver Dmc1 mRNA to spermatocytes, which recover the chromosome recombination as well as spermatogenesis.

In this study, we hypothesize that by rationally designing the structure of ionizable lipids, LNPs can deliver mRNAs encoding functional proteins into spermatocytes to restore spermatogenesis caused by genetic defects. Based on prior research findings, we integrated the above three bioactive units and designed a series of cholesterol‐amino‐phosphate (CAP) lipids, which can be formulated into CAP LNPs (**Figure** **1**). These CAP lipids consist of two rigid hydrophobic tails (cholesterol molecules), a phosphate linker, and an amino head. We hypothesize that this architecture can promote phase transformation, endosome escape and the release of the payloads. In terms of animal model, we chose DNA Meiotic Recombinase 1 (Dmc1) gene knockout (Dmc1^−/−^) mice as therapeutic model due to its similar phenotypes to azoospermia in humans.^[^ ^11^ ^]^ More importantly, Dmc1 mutation can lead to meiotic arrest as the Dmc1 protein plays a central role in meiosis by forming homologous recombination (HR) and repairing DNA breaks. Therefore, restoration of spermatogenesis in Dmc1^−/−^ mice has strong clinical implications for the treatment of human azoospermia characterized by meiotic arrest. As shown in Figure 1, we encapsulated mRNA encoding Dmc1 protein and microinjected into seminiferous tubules of Dmc1^−/−^ mice through rete testis. Subsequently, CAP LNPs can release mRNAs to the cytosol, which are translated to Dmc1 proteins. The sufficient supplementation of Dmc1 protein can rescue the chromosomes recombination, thereby recovering the meiosis and spermatogenesis.

## 2 Results and Discussion

### 2.1 Synthesis, Formulation, Characterizations of CAP LNPs for mRNA Delivery

**Figure 2:**
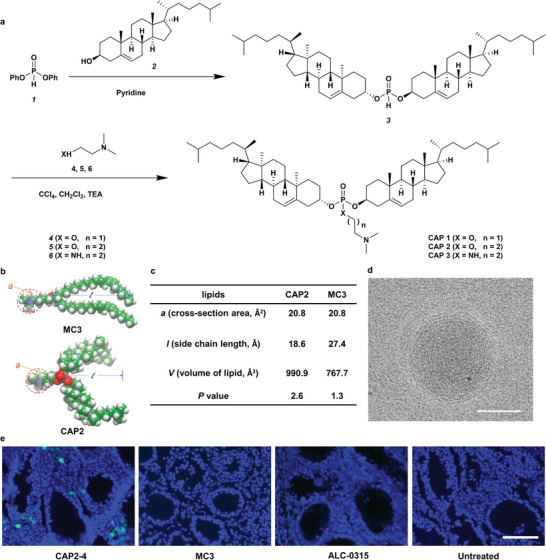
Synthesis of CAP lipids and delivery of mRNA using CAP LNPs. a) Synthesis and structures of CAP compounds. b) 3D structures of CAP2 and MC3 lipids. c) Calculated parameters and *P* values of CAP2 and MC3 lipids. d) Representative Cryo‐EM image of CAP2‐4 LNPs. Scale bar, 50 nm. e) Representative fluorescence microscopy images of seminiferous tubules from CAP2‐4 LNPs, MC3 LNPs, and ALC‐0315 LNPs administrated mice in comparison to untreated mice. Scale bar: 50 µm.

First, we constructed a synthetic route to CAP derivatives (**Figure** **2**a), which consist of two cholesterols, one phosphate linker, and an amino group. Briefly, dicholesterol phosphite (compound 3) was obtained through a transesterification reaction of diphenyl phosphite and cholesterol. Then, compound 3 underwent an Atherton–Todd reaction with amines or amino alcohols in the presence of carbon tetrachloride to afford the corresponding phosphoramidate or phosphotriester, respectively (Figure 2a).^[^ ^12^ ^]^ The structures of CAPs were confirmed by ^1^H NMR and mass spectrum (MS) (Figures S1–S4, Supporting Information). Next, we formulated CAP LNPs with the following components: CAPs, dioleoyl phosphatidylethanolamine (DOPE), cholesterol, 1,2‐dimyristoyl‐rac‐glycero‐3‐methoxypolyethylene glycol‐2000 (DMG‐PEG2000), and firefly luciferase mRNA as we reported previously (Figure S5, Supporting Information).^[^ ^8^, ^13^ ^]^ The CAP LNPs showed particle size from 100 to 150 nm with PDI lower than 0.3 (Figure S6, Supporting Information). We then investigated the mRNA delivery efficiency of CAP LNPs in Hep3B cells in vitro. D‐Lin‐MC3‐DMA also known as MC3, is an FDA‐approved ionizable amide lipid that has been used as a lipid component in ONPATTRO. In this study, MC3 based LNPs were included as a control group. CAP2‐4 LNPs showed the highest luminescence intensity among all the formulations (Figure S7, Supporting Information), which was around twice the luminescence intensity of MC3 LNPs.

As reported in the literature,^[^ ^10^ ^]^ the geometry of the ionizable lipids, which can be described by *P* value, can greatly affect the delivery efficiency of the LNPs. Typically, lipids with a large *P* value are favorable to form an inverted conical shape, which facilitates the inverted hexagonal (H_II_ phase) transformation of the endosome membrane and the endosomal escape of payloads.^[^ ^10^ ^]^ Hence, we calculated the *P* value of CAP2 and MC3, which can predict the nanostructure formed by the lipids. In general, lipids of small *P* values (<1/3) would aggregate into spheres. Lipids of medium *P* values would pack into hexagonal (H_I_ phase) (1/3≤P≤1/2) or planar (1/2<P≤1) nanostructures. Large *P* values (>1) that correspond to “a small head with large tails” would lead to H_II_ phase or reversed spherical nanostructures. According to the equation reported before,^[^ ^14^ ^]^ *P* value for a lipid molecule is calculated as: *P* = *v/(ℓa)*, where *a* is head area, and *v* and *ℓ* are the tail volume and length, respectively. Our calculated *P* value of CAP2 is 2.6, which is much higher than that of MC3 (*P* = 1.3) (Figure 2b,c). In general, the formation of the H_II_ phase is thought to be more likely to induce the rupture of the endosomal membrane and the release of RNA payloads into cytosol.^[^ ^10^ ^]^ Therefore, CAP2 with a *P* value of 2.6 is more likely to assemble into a H_II_ phase nanostructure and facilitate the intracellular mRNA delivery, which supports our design of the CAP molecules.

Based on these results, we selected CAP2‐4 LNPs for the following studies and further characterized their physicochemical properties. The size of CAP2‐4 LNPs was 126.4 ± 5.7 nm with a PDI of 0.24 ± 0.01 (Figure S8a,b, Supporting Information). CAP2‐4 LNPs displayed a slightly positive charge, and the mRNA encapsulation efficiency was around 90%. Cryo‐electron microscopy (cryo‐EM) showed that CAP2‐4 LNPs were spherical and multi‐layered particles with a size around 100 nm, which was consistent with the dynamic light scattering (DLS) measurement (Figure 2d; Figure S9, Supporting Information). In addition, we conducted a calcein assay to visualize the endosomal escape of CAP2‐4 LNPs. Calcein is a membrane‐impermeable fluorescent indicator that is normally trapped in endosomes. Upon permeabilization of the endosomal membrane, released calcein showed a diffuse fluorescent signal, indicating endosomal escape.^[^ ^13^ ^]^ In our experiment, we observed diffused green fluorescence in the cytoplasm when we treated Hep3B cells with both calcein and CAP2‐4 LNPs. In the control group with only calcein incubated cells, the fluorescent signals were shown in scattered dots. These results suggest the rupture of endosomal compartments following CAP2‐4 LNPs treatment (Figure S10, Supporting Information). To study the endocytic pathway of CAP2‐4 LNPs, Hep3B cells were incubated with endocytosis inhibitors including 5‐(*N*‐ethyl‐*N*‐isopropyl) amiloride (EIPA), chlorpromazine (CPZ), and methyl‐*β*‐cyclodextrin (M*β*CD) to inhibit the macropinocytosis, clathrin, and caveolae endocytic pathways, respectively. CAP2‐4 LNPs encapsulated with Alexa‐Fluor‐647‐labeled RNA were then added to the pretreated cells. We found that the cellular uptake of CAP2‐4 LNPs was significantly reduced by 26% and 43% after incubation by M*β*CD and CPZ, respectively, indicating that the uptake of CAP2‐4 LNPs is mediated by both clathrin and caveolae pathways tested in this study (Figure S11, Supporting Information).

Next, we applied CAP2‐4 LNPs to deliver the green fluorescent protein (GFP) mRNA as a reporter in a Dmc1^−/−^ mouse model. CAP2‐4 LNPs were microinjected to seminiferous tubules and expression of GFP was analyzed 24 h after administration. Two clinically used LNPs, MC3 LNPs and ALC‐0315 LNPs, were administered in the same way as the controls. As shown in Figure 2e, CAP2‐4 LNPs induced dramatic green fluorescence around the seminiferous tubules, while the GFP signals in MC3 LNPs and ALC‐0315 LNPs group were barely detected. These results demonstrated that CAP2‐4 LNPs were superior vehicles for mRNA delivery in spermatocytes. Based on these results, we selected CAP2‐4 LNPs for the following studies.

### 2.2 CAP LNPs can Deliver saRNA to Spermatocytes in a Dmc1^−/−^ Mouse Model

**Figure 3:**
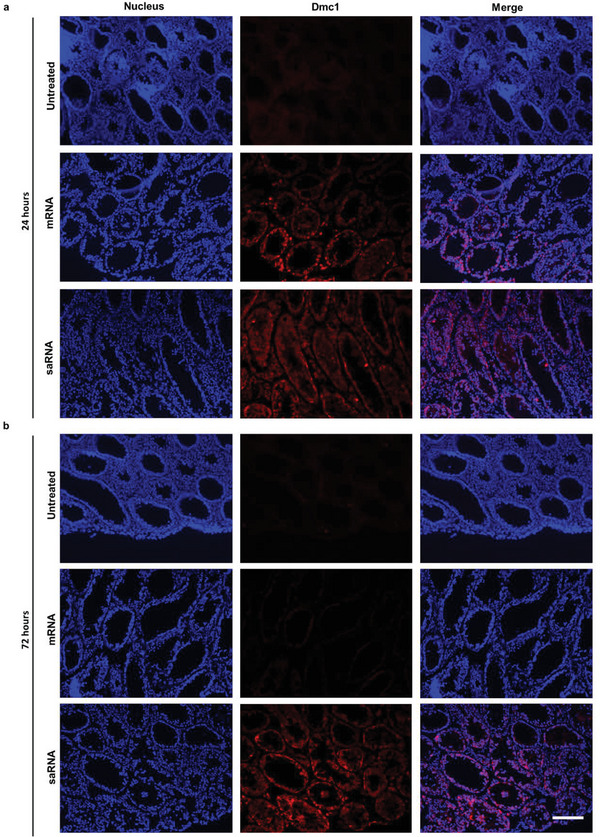
Delivery of traditional mRNA or saRNA encoding Dmc1 protein in Dmc1^−/−^ mice. Fluorescence microscopy of representative seminiferous tubules a) 24 h or b) 72 h after administration with CAP2‐4 LNPs encapsulating FLAG‐tagged Dmc1 mRNA and FLAG‐tagged Dmc1 saRNA in comparison to the untreated group. Scale bar: 50 µm.

Unlike traditional mRNA, self‐amplifying mRNA (saRNA, also named replicon RNA) encodes not only the proteins of interest but also proteins that enable intracellular RNA amplification.^[^ ^15^ ^]^ Thus, saRNA can induce the expression of desired proteins for a longer period compared to traditional mRNA.^[^ ^15^, ^16^ ^]^ To validate the ability of CAP LNPs to deliver saRNA in vivo, we encapsulated firefly luciferase saRNA into CAP2‐4 LNPs and injected them into seminiferous tubule of Dmc1^−/−^ mice. As shown in Figure S12 (Supporting Information), the luminescence intensity on the injected side (left testis) continued to increase for 10 days, demonstrating that CAP2‐4 LNPs can efficiently deliver saRNA into testis and achieve sustained protein expression. We then compared the expression time course of Dmc1 protein between traditional mRNA and saRNA. We encapsulated Flag‐tagged Dmc1 mRNA or saRNA into CAP2‐4 LNPs and injected both formulations into seminiferous tubule of Dmc1^−/−^ mice. 24 and 72 h after administration, Dmc1 expression in seminiferous tubules was evaluated by immunofluorescence staining. As shown in **Figure** **3**a, obvious Dmc1 expression was observed in both CAP2‐4 LNP‐mRNA and CAP2‐4 LNP‐saRNA treated mice on 24 h, but not in untreated mice. 72 h after the administration of the CAP2‐4 LNP‐mRNA, there was almost no fluorescence signal in the seminiferous tubules (Figure 3b). On the contrary, the CAP2‐4 LNP‐saRNA‐treated group still displayed a strong fluorescence signal (Figure 3b). These results indicated that the delivery of saRNA via CAP2‐4 LNP can maintain the Dmc1 protein expression for at least three days. In male mice, it takes around 3 days from the initiation of prophase I to pachytene spermatocytes. During this period, Dmc1 is required for chromosome recombination,^[^ ^17^ ^]^ thus we chose the formulation of CAP2‐4 LNP‐saRNA for further functional studies.

### 2.3 Delivery of saRNA using CAP LNPs Restores Dmc1 Protein Function

**Figure 4:**
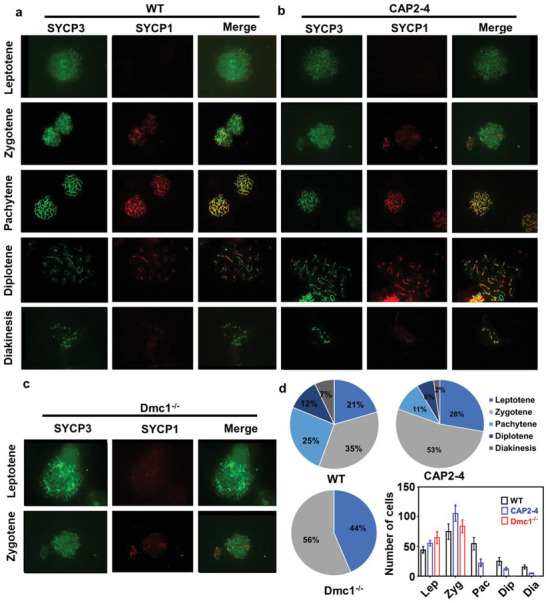
Delivery of saRNA using CAP LNPs restores the Dmc1 function in Dmc1^−/−^mice. Chromosome spreading assay of spermatocytes from a) wild‐type (WT) mice; b) CAP2‐4 Dmc1 saRNA LNPs treated Dmc1^−/−^ mice; and c) untreated Dmc1^−/−^ mice. SC was categorized by staining chromosomes with anti‐SYCP3 antibody (green) and SYCP1 antibody (red). d) Percentage of spermatocytes in different substages are indicated in the pie charts and numbers of spermatocytes at each substage are indicated in a column chart. All data are presented as mean ± s.d.

Subsequently, we examined the function of Dmc1 protein by analyzing synaptonemal complex (SC). SC is a specific structure formed during meiosis I and is responsible for the synapsis of homologous chromosomes. Since Dmc1 deficiency can disrupt the SC formation and arrest the spermatogenesis in the late prophase of Meiosis I, SC is considered a critical indicator of Dmc1 protein function recovery.^[^ ^18^ ^]^ The Dmc1^−/−^ mice were treated with CAP2‐4 LNP‐saRNA. On day 4 after treatment, the testes were collected. The SC in the spermatocytes was examined by immunofluorescence staining of SYCP1 and SYCP3 (two main constitutes involved in SC formation). As shown in **Figure** **4**a, SYCP3 and SYCP1 staining clearly showed the characteristic patterns of the five substages including leptotene, zygotene, pachytene, diplotene, and diakinesis in WT mice.^[^ ^19^ ^]^ In contrast, the SC from Dmc1^−/−^ mice was arrested in the early stage of prophase and the SC of pachytene, diplotene, and diakinesis stages was not observed (Figure 4c). In the CAP2‐4 LNP‐saRNA treated group, we observed the SC in the pachytene stage, which indicated the restoration of synapsis. In addition, the observation of SC in the diplotene and diakinesis stages further confirmed that SC can disassemble and transit to metaphase (Figure 4b). As indicated by Figure 4d, the percentages of spermatocytes in the stages of pachytene, diplotene, and diakinesis increased to 11%, 6%, and 2%, respectively after the CAP2‐4 LNP‐saRNA treatment. The number of SC in the last three substages (pachytene, diplotene, and diakinesis stages) was significantly higher than Dmc1^−/−^ mice. Collectively, these results demonstrated that the delivery of saRNA using CAP LNPs restored the function of Dmc1 protein in spermatocytes.

### 2.4 CAP LNP‐saRNA Rescues the Spermatogenesis in Dmc1^−/−^ Mice

**Figure 5:**
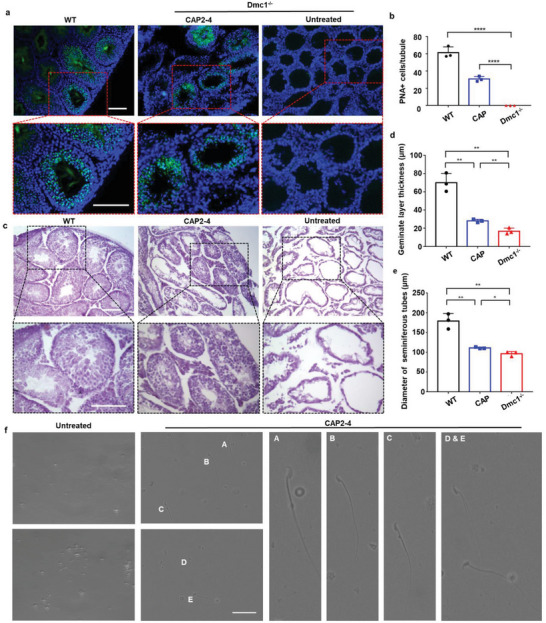
Delivery of saRNA using CAP LNPs rescues the infertile phenotype of Dmc1^−/−^ mice. a) Fluorescent images of PNA‐lectin labeled spermatozoa of WT; Dmc1^−/−^ mice treated with CAP2‐4 LNPs, and untreated Dmc1^−/−^ mice. Scale bar: 50 µm. b) The mean number of PNA positive cells per seminiferous tubules of WT mice, Dmc1^−/−^ mice treated with CAP2‐4 LNPs and Dmc1^−/−^ mice. c) Histological analysis of WT; Dmc1^−/−^ mice treated with CAP2‐4 LNPs, and untreated Dmc1−/− mice. The black box indicates images at magnifications of 20× . Scale bar: 50 µm. d) The average geminate layer thickness of WT; Dmc1^−/−^ mice treated with CAP2‐4 LNPs, and untreated Dmc1^−/−^ mice. e) The diameter of seminiferous tubes of WT; Dmc1^−/−^ mice treated with CAP2‐4 LNPs, and untreated Dmc1^−/−^ mice. f) Representation of sperms from Dmc1^−/−^ mice treated with CAP2‐4 LNPs (a)–(e), and microscopy analysis of untreated Dmc1^−/−^ mice. Scale bar: 20 µm. All data are representative images from *n*  =  3 independent samples and all the statistical analysis are presented as mean ± s.d. (*n* = 3). Statistical significance was analyzed by one‐way ANOVA with Bonferroni's multiple comparisons test. *P < 0.05, **P < 0.01, ****P < 0.0001.

Finally, we investigated whether the restoration of Dmc1 protein by CAP2‐4 LNP‐saRNA could rescue the infertility of Dmc1^−/−^ mice. The Dmc1^−/−^ mice were treated with CAP2‐4 LNP‐saRNA. On day 10 after treatment, the testes and epididymis were collected. The sections of testes and epididymis were stained with Peanut Agglutinin (PNA)‐lectin, which can specifically bind to spermatids acrosomes (**Figure** **5**a; Figures S13 and S14, Supporting Information). PNA‐stained spermatids were localized in the seminiferous tubules and epididymal ducts of WT mice. However, no cells in the testes and epididymis of Dmc1^−/−^ mice were stained with PNA‐lectin, indicating a complete loss of spermatids production. In contrast, we observed the PNA‐stained cells in the seminiferous tubules and epididymal ducts in the CAP2‐4 LNP‐saRNA treatment group which is direct evidence for rescuing the infertile phenotype of Dmc1^−/−^ mice. The statistical analysis showed that the number of PNA‐positive cells per tubule in the treatment group was significantly higher than that in the untreated group and reached about one‐half of the WT group (Figure 5b). Therefore, these results proved that delivery of saRNA using CAP LNPs can effectively rescue spermatids production in Dmc1^−/−^ mice.

Additionally, histological analysis of testis samples was performed to evaluate spermatogenic development. As shown in Figure 5c and Figure S15 (Supporting Information), WT mice exhibited normal testicular morphology, while Dmc1^−/−^ mice displayed an arrest of spermatogenesis at the spermatocyte stage and completely lacked postmeiotic cells. Compared with WT mice, the thickness of the germinal layer and the diameter of the seminiferous tubes were significantly reduced in Dmc1^−/−^ mice (Figure 5d,e). In contrast, CAP2‐4 LNP‐saRNA re‐established the testicular morphology of Dmc1^−/−^ mice with increased germinate layer thickness and diameter of seminiferous tubes (Figure 5d,e). At high magnification (100×), we observed spermatids in CAP2‐4 LNP‐saRNA treated mice (Figure S16, Supporting Information). We also incubated the epididymis from CAP2‐4 LNP‐saRNA treated and untreated Dmc1^−/−^ mice in Human Tubal Fluid (HTF) medium for 20 min to release sperms. The cytology smears of the sperm suspension showed that the epididymis of the CAP2‐4 LNP‐saRNA treated group produced sperms with oval‐shaped head and an uncurled single tail, while no sperm‐like cell was observed in the untreated group (Figure 5f; Figure S17, Supporting Information). We also observed sperms with progressive motility which swim in a straight line or large circle which demonstrate their mobility (Movies S1 and S2, Supporting Information). To further evaluate the fertility of the CAP2‐4 LNP‐saRNA treated mice, we collected the sperms on day 10 after treatment via an intracytoplasmic sperm injection (ICSI). We microinjected 21 sperms into wild type C57Bl6 mouse oocytes and observed 12 embryos developed to the 2‐cell stage on day 2 after ICSI. On day 5 after ICSI, we observed 3 embryos proceeded to morula stage. Overall, these results indicated that delivery of saRNA using CAP LNPs can partially rescue the infertility of Dmc1^−/−^ mice. Considering the severity of infertility caused by Dmc1 deficiency, the restoration of the infertile phenotype of Dmc1^−/−^mice validated the effectiveness of CAP LNPs to deliver saRNA in spermatocytes. In particular, the production of spermatids in testes and sperms in epididymis highlights the potential of this therapeutic strategy in the treatment of male infertility in combination with the ART.

## 3 Conclusions

Effective mRNA delivery to spermatocytes represents a potential therapy for male infertility. To develop an efficient delivery system in this cell population, we carefully conceived the bioactive components including cholesterol, amino group, and a phosphate linker. Furthermore, we designed a chemical scaffold with a *P* value much higher than 1, which provides a H_II_ phase, a favorable nanostructure for the formulated CAP LNPs and facilities the mRNA release from the endosome. In particular, CAP2‐4 LNPs showed much more efficient mRNA delivery than MC3 LNPs both in vitro and in vivo. Moreover, we identified an optimal formulation of CAP LNPs formulated from three components (CAP2, DOPE, and DMG‐PEG) without the extra addition of cholesterol, which simplified the nanoparticle composition. In a mouse infertility model, we showed that by using the newly developed CAP LNPs to deliver the saRNA encoding the Dmc1 protein, the functional protein was restored and infertility of the Dmc1^−/−^ mouse model was rescued consequently. Overall, this work demonstrates the feasibility of mRNA‐based therapy in the treatment of male reproductive disorders caused by genetic mutations. As far as we know, this is the first time to apply saRNA for treating genetic diseases other than immunotherapy. In terms of the clinical translation, remarkable strides have been made in the rete testis microinjection and aspiration techniques. For example, ultrasound‐guided rete testis flushing, and aspiration technique has entered the clinical trial for the treatment of nonobstructive azoospermia (NCT03291522). With these advances, this LNPs‐saRNA system can be further developed as an effective method to investigate male infertility and used clinically as a complement of ART to benefit a wide range of infertility.

## 4 Experimental Section

### Materials and Reagents

DOPE, cholesterol, and DMG‐PEG2000 were purchased from Avanti Polar Lipids Inc. (Alabaster, AL). Eagle's minimum essential medium (EMEM) and other cell culture supplies were purchased from Corning Incorporated (Corning, NY). Quant‐iT RiboGreen RNA reagent and Gibco heat‐inactivated fetal bovine serum (FBS) were purchased from Thermo Fisher Scientific (Waltham, MA). All the other chemical reagents were obtained from Sigma‐Aldrich or Abcam and used without further purification.

### Preparation and Characterization of LNPs

The synthesis of CAPs was described in the Supporting Information. The mRNA and replicon were synthesized following the previously reported method.^[^ ^8a^ ^]^ The LNP formulations were prepared based on the previous reports.^[^ ^13^ ^]^ Briefly, the ethanol phase was composed of CAP lipids MC3, or ALC‐0315 with other helper lipids dissolved in ethanol at a certain molar ratio; Aqueous phased was prepared by diluting firefly luciferase (FLuc) mRNA, Dmc1 mRNA, or saRNA in a citrate buffer (pH 3). The LNPs were prepared by a rapid mixing method via a pipetting technique or a microfluidic device and were then purified by dialysis. Particle size and zeta potential were quantified by NanoZS Zetasizer (Malvern). The entrapment efficiency was measured by the RiboGreen assay. The morphology of LNPs was characterized by a Cryo‐EM (Thermo Scientific Glacios) device as we described previously.^[^ ^13^ ^]^

### *P* Value Calculation

Assuming that an amphiphilic molecule that has a head of an area *a*, a volume *v* and a tail length *ℓ* inside an aggregate, the dimensionless packing parameter *P* is defined as: *P* = *v/(ℓa)*. 2D structures of both compounds were converted to 3D coordinates using OpenBabel^[^ ^20^ ^]^ and the MMFF94 force field.^[^ ^21^ ^]^ The generated 3D structures were optimized using Gaussian 16^[^ ^22^ ^]^ at the HF/6‐31G* level. From the energy‐minimized structure, *a* was measured as the cross‐section area of the head group, and *ℓ* was measured as the distance between the center of mass (COM) of the ester linker group and the terminal methyl groups for MC3 or between the phosphorus atom and the terminal methyl groups for CAP2 (Fig. 2b). The van der Waals volume was calculated using an approximate method termed Atomic and Bond Contributions of van der Waals volume (VABC)^[^ ^23^ ^]^ that was shown to reproduce the molecular volume calculated with more accurate method such as the Voronoi tessellation approach:^[^ ^24^ ^]^ *V*_vdW_ *= Σ*_all atom  contributions_ − 5.92*N*_B_ − 14.7*R*_A_ − 3.8*R*_NA_, where *N*_B_ is the number of bonds, *R*_A_ is the number of aromatic rings, and *R*_NA_ is the number of nonaromatic rings.

### Cell Culture and In Vitro Luciferase Assay

Hep3B cells were cultured in Eagle's minimum essential medium (Corning) with 10% fetal bovine serum (FBS). Before treated by CAP LNPs, cells were seeded at a density of 2 × 10^4^ cells per well on a white 96‐well flatbottom plate overnight. The dose was 50 ng Firefly luciferase mRNA per well. After 18 h incubation, Bright‐Glo luciferase (Promega) was added, and the luminescence activity was determined by Cytation 5 (Biotek).

### Endosome Escape and Cell Uptake Assay

A total of 3 × 10^4^ Hep3B cells in 300 µL medium were plated in an imaging dish (Ibidi) and incubated at 37 °C in 5% CO_2_ incubator for 24 h. Calcein (final concentration 150 µg mL^−1^) was added to the cells alone or in combination with CAP2‐4 LNPs encapsulating with Firefly Luc mRNA and Alexa‐Fluor‐647‐labeled RNA at a 1:1 weight ratio. After 2 h of coincubation, the cells were imaged using Nikon A1R Live Cell confocal laser scanning microscope (Melville, NY, USA) and the acquired images were analyzed with NIS‐Elements AR (Version 5.20.00.). For cell uptake assay, Hep3B cells were seeded in a 24‐well plate at 10^5^ cells per well and cultured for 24 h. Then, the cells were treated by endocytic inhibitors EIPA, M*β*CD and CPZ to inhibit different endocytic pathways. After 30 min, these cells were treated by CAP2‐4 LNPs encapsulating with FLuc mRNA and Alexa‐Fluor 647‐labelled RNA at a 1:1 weight ratio. After 3 h incubation, cellular uptake was quantified by a flow cytometer (LSRII, BD).

### In Vivo Rete Testis Microinjection and Preparation of Histologic Sections

All animal experimental procedures were performed according to the guidelines approved by the Institutional Animal Care and Use Committee (IACUC) of the Ohio State University and complied with all relevant ethical regulations as applicable (2014A00000106‐R2). Heterozygous mice were bred together to maintain a live colony. The genotyping protocol was followed by Protocol 23 094: Standard PCR Assay – Dmc1<tm1Jcs>(JAX). The expected genotyping results were Mutant = 147 bp; Heterozygote = 147 and 233 bp; Wild type = 233 bp. All the Dmc1^−/−^ mice used in the study were 8–10 weeks. Germ Cells from the Dmc1^−/−^ mice were arrested in Early Prophase I. Testes of Dmc1^−/−^ mice were smaller than normal mice and no spermatozoa could be detected in the testes or epididymis. CAP LNPs were injected into the seminiferous tubules of mice through the previously reported microinjection method.^[^ ^25^ ^]^ For all the administration, the mRNA concentration is 0.24 mg mL^−1^, and the volume is 30 µL per testis. After administration, testes were harvested to prepare cryostat microtome section or meiotic chromosome spreads. For cryostat microtome section preparation, the testes were fixed by immersion in 4% paraformaldehyde in 0.1 m phosphate buffer (pH 7.4) at 4 °C for 4 h, then dehydrated in 30% sucrose, embedded in optimal cutting temperature compound (OCT compound), cut into 5‐µm‐thick sections using a microtome, and mounted onto glass slides. The preparation of meiotic chromosome spreads followed the previous literature.^[^ ^19^ ^]^ Briefly, the testes were incubated in a hypotonic solution to swell spermatocytes. Then spermatocytes are released into a sucrose solution to obtain a cell suspension, and nuclei were spread onto fixative‐soaked glass slides.

### Immunofluorescent Staining

First, the histologic sections were blocked in the donkey serum for 30 min. The primary antibody (dissolved in 1% BSA in 1 × TBS) was then added and the slides were incubated in a humid chamber at 4 °C overnight. After incubation, the slides were washed by 1 × TBS 3 times (5 min each time) and incubated with the secondary antibody (dissolved in 1% BSA in TBS) in the dark at room temperature. 1 h After incubation of secondary incubation, slides were washed three times with TBS in the dark (5 min each time) and mounted by antiquencher. Primary antibodies were diluted as follows: Anti‐Mouse SYCP3 Antibody (1:250); Anti‐Rabbit SYCP1 Antibody (1:250); Anti‐Rabbit‐FLAG Tag (1:100); Secondary antibodies were diluted as follows: Goat Anti‐Mouse IgG H&L‐Alexa Fluor 488 (1:500); Goat Anti‐Rabbit IgG H&L Alexa Fluor 555 (1:500); PNA lectin (1:200).

### Sperm Collection

Mice were euthanized and the caudal epididymites were removed and placed into the HTF medium. The epididymis was minced 4 to 6 times by forceps and scissors, and incubated at 37 °C, with 5% CO_2_ and air for 20 min to release the sperm into the medium. The tissue was removed, and the sperm suspension was used to prepare cytology smears.

### ICSI

Mice were sacrificed and the epididymites were collected in FERTIUP medium (COSMO BIO). The fat and blood of the cauda epididymites were removed completely. The epididymites were teared by forceps and scissors, incubated at 37 °C for 10 min to release the sperms. After the removing epididymal tissues, the sperm suspension was collected into tubes and stored at −20 °C before ICSI. ICSI was performed as described in Manipulating Mouse Embryo (4th edition). In brief, 6 weeks old C57Bl6 (Jackson lab, US) female mice were superovulated with 5IU PMS (Sigma) then 5IU HCG (Sigma) 48 h later. 15 h after HCG, oocytes were collected from the mouse oviduct. Meanwhile, CAP2‐4 treated mouse sperm head was separated from tail then injected into C57Bl6 oocyte by micromanipulator that was equipped with piezo impact drive unit (Prime Tech). M2‐embryo medium (Millipore) was used during ICSI. Oocytes injected with sperm head were incubated in KSOM (Millipore) media at 37 °C, with 5% CO_2_ to designed time periods.

## Conflict of Interest

Y.D. is a scientific advisory board member of Oncorus Inc, Arbor Biotechnologies, and FL85.

## Supporting information

Supporting Information

Supplemental Movie 1

Supplemental Movie 2

## Data Availability

The data that support the findings of this study are available from the corresponding author upon reasonable request.
