# Correction to Cholesterol‐Amino‐Phosphate (CAP) Derived Lipid Nanoparticles for Delivery of Self‐Amplifying RNA and Restoration of Spermatogenesis in Infertile Mice

**DOI:** 10.1002/advs.202412632

**Published:** 2024-10-22

Du S, Li W, Zhang Y, et al. Cholesterol‐Amino‐Phosphate (CAP) Derived Lipid Nanoparticles for Delivery of Self‐Amplifying RNA and Restoration of Spermatogenesis in Infertile Mice. *Advanced Science*, **2023**, *10*(11): 2300188.

In the original version of Figure 4, the red highlight in Figure 4b is incorrectly identical to that in Figure 4c. This is an error that occurred during sub‐figure assembly. The correct figure is presented below.

Original Figure 4


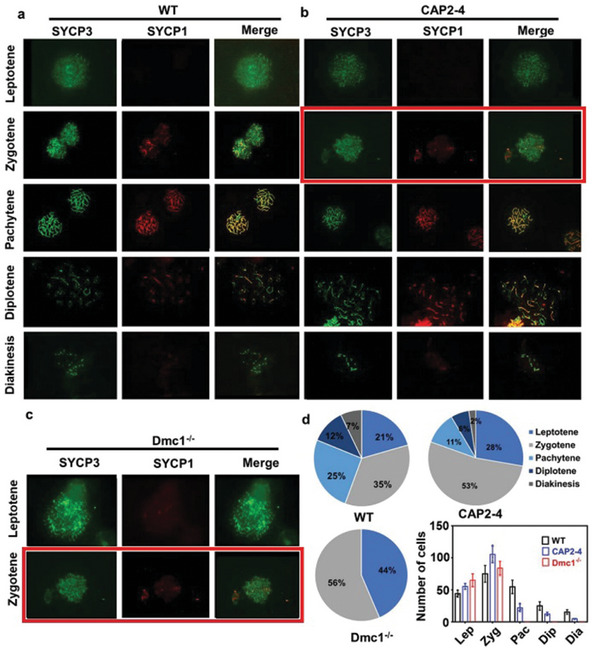


Corrected Figure 4


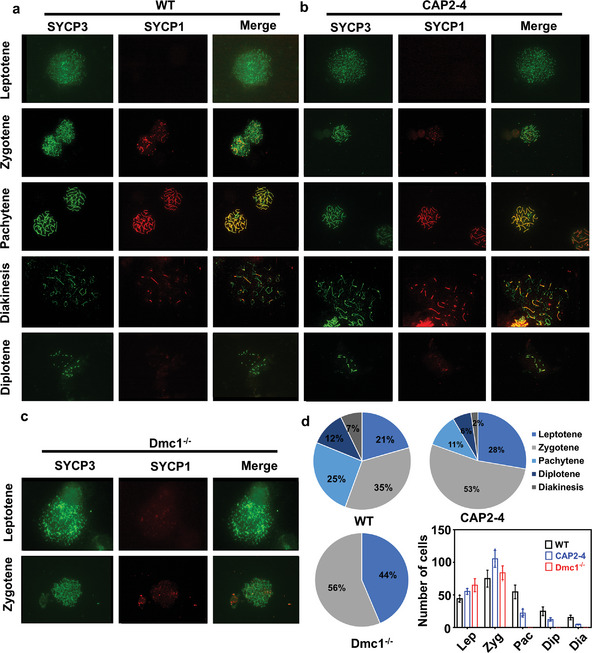


We apologize for this error.

