# Supplementary material for: Cholesterol‐Amino‐Phosphate (CAP) Derived Lipid Nanoparticles for Delivery of Self‐Amplifying RNA and Restoration of Spermatogenesis in Infertile Mice
Source: Adv Sci (Weinh). 2023 Feb 7;10(11):2300188. doi: 10.1002/advs.202300188 (PMC10104632; doi:10.1002/advs.202300188)
Supplement: Supplementary file 1 — Supporting Information [file ADVS-10-2300188-s002.pdf]

ADVANCED SCIENCE
Open Access

Supporting Information

for *Adv. Sci.*, DOI 10.1002/advs.202300188

Cholesterol-Amino-Phosphate (CAP) Derived Lipid Nanoparticles for Delivery of Self-Amplifying RNA and Restoration of Spermatogenesis in Infertile Mice

*Shi Du, Wenqing Li, Yuebao Zhang, Yonger Xue, Xucheng Hou, Jingyue Yan, Jeffrey Cheng, Binbin Deng, David W. McComb, Jennifer Lin, Hong Zeng, Xiaolin Cheng, Darrell J. Irvine, Ron Weiss and Yizhou Dong**

Cholesterol-amino-phosphate (CAP) derived lipid nanoparticles for delivery of self-amplifying RNA and restoration of spermatogenesis in infertile mice

Shi Du[1,13], Wenqing Li[1,13], Yuebao Zhang[1,13], Yonger Xue[1], Xucheng Hou[1], Jingyue Yan[1], Jeffrey Cheng[1], Binbin Deng[2], David W. McComb[2,3], Jennifer Lin[4], Hong Zeng[4], Xiaolin Cheng[5], Darrell J. Irvine[6,7,8,9,10], Ron Weiss[6,7], Yizhou Dong[1,11,12*]

[1]Division of Pharmaceutics & Pharmacology, College of Pharmacy, The Ohio State University, Columbus, OH, USA.[2]Center for Electron Microscopy and Analysis, The Ohio State University, Columbus, OH, USA. [3]Department of Materials Science and Engineering, The Ohio State University, Columbus, OH, USA. [4]Transgenic, Knockout, and Tumor Model Center, Stanford University School of Medicine, Stanford, CA, USA. [5]Division of Medicinal Chemistry and Pharmacognosy, College of Pharmacy, The Ohio State University, OH, USA.[6]Department of Biological Engineering, Massachusetts Institute of Technology, Cambridge, MA, USA. [7]Koch Institute for Integrative Cancer Research, Massachusetts Institute of Technology, Cambridge, MA, USA. [8]Department of Materials Science and Engineering, Massachusetts Institute of Technology, Cambridge, MA, USA. [9]Ragon Institute of Massachusetts General Hospital, Massachusetts Institute of Technology and Harvard University, Cambridge, MA, USA. [10]Howard Hughes Medical Institute, Chevy Chase, MD, USA. [11]Department of Biomedical Engineering, Center for Clinical and Translational Science, Comprehensive Cancer Center, Dorothy M. Davis Heart & Lung Research Institute, Department of Radiation Oncology, Center for Cancer Engineering, Center for Cancer Metabolism, Pelotonia Institute for Immune-Oncology, The Ohio State University, Columbus, OH, USA. [12] Icahn Genomics Institute, Precision Immunology Institute, Department of Oncological Sciences, Tisch Cancer Institute, Friedman Brain Institute, Icahn School of Medicine at Mount Sinai, New York, NY, USA. [13]These authors contributed equally: Shi Du, Wenqing Li, Yuebao Zhang. *e-mail: yizhou.dong@mssm.edu

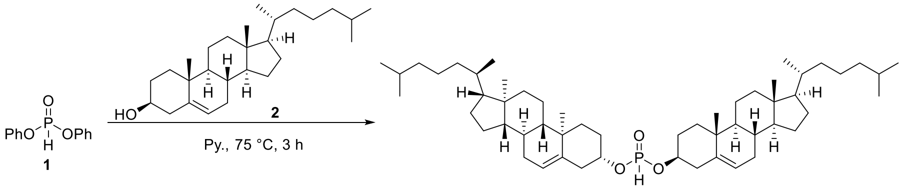

Supplementary Figure 1. Synthesis of 3. To a solution of diphenyl phosphonate **1** (0.7 g, 3.0 mmol) in 3.0 mL of pyridine was added cholesterol **2** (2.38 g, 6.15 mmol). The resulting solution was then allowed to warm to 75 °C and stirred for 3 h. Pyridine was removed under reduced pressure, the residue was diluted with 100 mL of DCM and washed with 10 mL of 1 N aqueous NaOH solution and 10 mL of water. The organic phase was dried over anhydrous Na_2SO_4, filtered, and the solvent was removed under reduced pressure. The residue was purified by silica gel chromatography (0%–10% Ethyl acetate in Hexane). 2.0 g of compound **3** was obtained as a white powder, yield 81.4%. ^{1}H NMR (300 MHz, Chloroform-*d*) δ 6.89 (d, *J* = 688.8 Hz, 1H), 5.38 (d, *J* = 5.1 Hz, 2H), 4.27 (ddt, *J* = 15.3, 10.8, 5.4 Hz, 2H), 2.52 - 2.38 (m, 4H), 1.98 (ddd, *J* = 13.5, 6.9, 2.7 Hz, 6H), 1.89 – 1.79 (m, 4H), 1.78 – 1.66 (m, 2H), 1.58 (dd, *J* = 7.2, 4.5 Hz, 2H), 1.55 – 1.40 (m, 10H), 1.39 – 1.23 (m, 8H), 1.23 – 1.04 (m, 14H), 1.04 (s, 2H), 1.01 (s, 6H), 0.97 (d, *J* = 1.9 Hz, 2H), 0.93 (s, 2H), 0.91 (d, *J* = 6.6 Hz, 6H), 0.86 (dd, *J* = 6.6, 1.5 Hz, 12H), 0.67 (s, 6H).

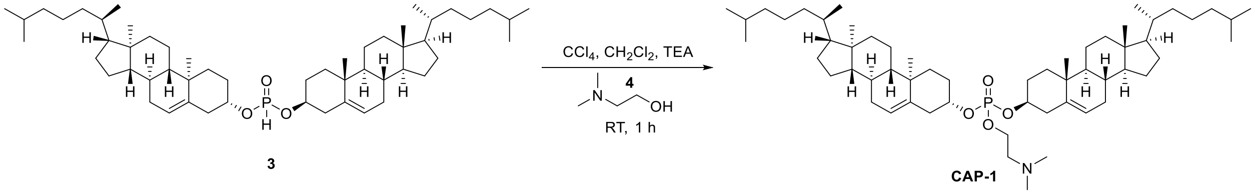

Supplementary Figure 2. Synthesis of CAP-1. To a flame-dried flask containing **3** (286.8 mg, 0.35 mmol) and carbon tetrachloride (2.0 mL) was added dropwise a solution of trimethylamine (194.6 µL, 1.4 mmol), DMAP (4.3 mg, 0.035 mmol), and **4** (178.2 mg, 2.0 mmol) in 1.0 mL of dry DCM under vigorous stirring at RT. The reaction mixture was stirred for 1 h, diluted with 50 mL of DCM, and washed three times with 50 mL of brine. The organic phase was isolated, dried over anhydrous Na_2SO_4, filtered, and the solvent was removed in vacuo. The residue was purified via silica gel chromatography (0% - 20% [mixture of 3% NH_4OH, 22% MeOH in dichloromethane] in dichloromethane). 188 mg of CAP-1 was obtained as a white powder, yield 59.3%. ^{31}P NMR (121 MHz, $CDCl_3$) δ -2.27. ^{1}H NMR (300 MHz, Chloroform-*d*) δ 5.36 (dd, *J* = 4.5, 2.7 Hz, 2H), 4.30 – 4.16 (m, 2H), 4.10 (dt, *J* = 7.2, 6.0 Hz, 2H), 2.61 (t, *J* = 6.0 Hz, 2H), 2.43 (d, *J* = 6.9 Hz, 4H), 2.29 (s, 6H), 1.99 (ddt, *J* = 13.8, 10.8, 4.2 Hz, 6H), 1.89 – 1.64 (m, 6H), 1.63 – 1.39 (m, 12H), 1.38 – 1.22 (m, 8H), 1.22 – 1.04 (m, 14H), 1.02 (d, *J* = 3.3 Hz, 2H), 1.00

(s, 6H), 0.96 (d, *J* = 3.3 Hz, 2H), 0.94 (s, 2H), 0.91 (d, *J* = 6.6 Hz, 6H), 0.86 (dd, *J* = 6.6, 1.5 Hz, 12H), 0.67 (s, 6H). MS (*m/z*): $[M+H]^+$ calcd. For For $C_{58}H_{101}NO_4P$, 906.7; found: 906.8.

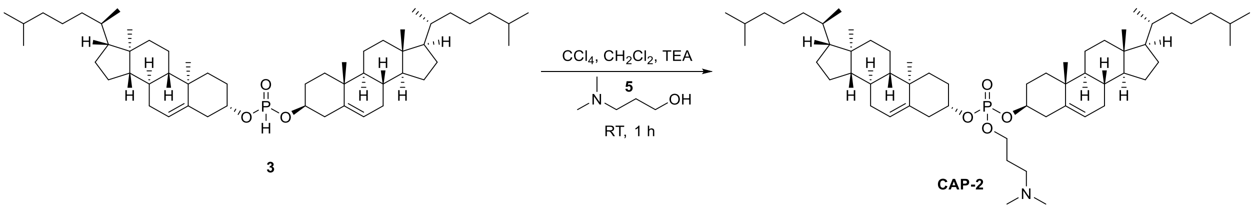

Supplementary Figure 3. Synthesis of CAP-2. To a flame-dried flask containing **3** (286.8 mg, 0.35 mmol) and carbon tetrachloride (2.0 mL) was added dropwise a solution of trimethylamine (194.6 µL, 1.4 mmol), DMAP (4.3 mg, 0.035 mmol), and **5** (144.5 mg, 1.4 mmol) in 1.0 mL of dry DCM under vigorous stirring at RT. The reaction mixture was stirred for 1 h, diluted with 50 mL of DCM, and washed three times with 50 mL of brine. The organic phase was isolated, dried over anhydrous Na_2SO_4, filtered, and the solvent was removed in vacuo. The residue was purified via silica gel chromatography (0% - 20% [mixture of 3% NH_4OH, 22% MeOH in dichloromethane] in dichloromethane). 196 mg of CAP-1 was obtained as a white powder, yield 60.8%. ^{31}P NMR (121 MHz, $CDCl_3$) δ -2.34. ^1H NMR (300 MHz, Chloroform-*d*) δ 5.43 – 5.34 (m, 2H), 4.27 – 4.11 (m, 2H), 4.07 (q, *J* = 6.6 Hz, 2H), 2.41 (dd, *J* = 12.0, 7.5 Hz, 6H), 2.24 (s, 6H), 2.06 – 1.91 (m, 6H), 1.83 (qd, *J* = 9.0, 8.1, 3.0 Hz, 6H), 1.76 – 1.64 (m, 2H), 1.62 – 1.40 (m, 12H), 1.39 – 1.23 (m, 8H), 1.22 – 1.03 (m, 14H), 1.03 (s, 2H), 1.00 (s, 6H), 0.96 (d, *J* = 3.6 Hz, 2H), 0.91 (d, *J* = 6.6 Hz, 6H), 0.86 (dd, *J* = 6.6, 1.4 Hz, 12H), 0.67 (s, 6H). MS (*m/z*): $[M+H]^+$ calcd. For $C_{59}H_{103}NO_4P$, 920.8; found: 920.9.

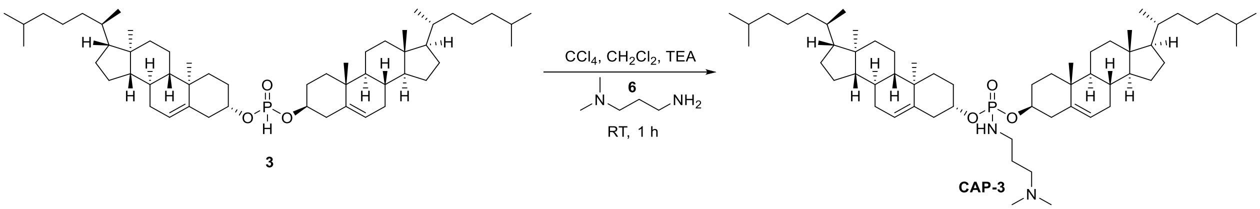

Supplementary Figure 4. Synthesis of CAP-3. To a flame-dried flask containing **3** (286.8 mg, 0.35 mmol) and carbon tetrachloride (2.0 mL) was added dropwise a solution of trimethylamine (194.6 µL, 1.4 mmol), DMAP (4.3 mg, 0.035 mmol), and **6** (143.1 mg, 1.4 mmol) in 1.0 mL of dry DCM under vigorous stirring at RT. The reaction mixture was stirred for 1 h, diluted with 50 mL of DCM, and washed three times with 50 mL of brine. The organic phase was isolated, dried over anhydrous Na_2SO_4, filtered, and the solvent was removed in vacuo. The residue was purified via silica gel chromatography (0% - 20% [mixture of 3% NH_4OH, 22% MeOH in dichloromethane] in dichloromethane). 189 mg of CAP-1 was obtained as a white powder, yield 58.7%. ^{31}P NMR (121 MHz, $CDCl_3$) δ 7.59. ^1H NMR (300 MHz, Chloroform-*d*) δ 5.36 (dt, *J* = 5.1, 2.7 Hz, 2H), 4.14 (h, *J* = 5.4 Hz, 2H), 3.43 (dt, *J* = 11.1, 6.6 Hz, 1H), 2.99 (dq, *J* = 9.0, 6.6

Hz, 2H), 2.52 – 2.32 (m, 6H), 2.22 (s, 6H), 1.99 (ddd, *J* = 16.5, 9.6, 6.3 Hz, 6H), 1.82 (tt, *J* = 12.6, 4.8 Hz, 6H), 1.69 – 1.40 (m, 16H), 1.40 – 1.23 (m, 8H), 1.23 – 1.05 (m, 14H), 1.03 (s, 2H), 1.00 (s, 6H), 0.97 (d, *J* = 3.0 Hz, 2H), 0.94 (s, 2H), 0.91 (d, *J* = 6.6 Hz, 6H), 0.86 (dd, *J* = 6.6, 1.5 Hz, 12H), 0.67 (s, 6H). MS (*m/z*): $[M+H]^+$ calcd. For $C_{59}H_{104}N_2O_3P$, 919.8; found: 919.9.

Name	CAP lipid	DOPE	Cholesterol	DMG-PEG
CAP1-1	20	30	40	0.75
CAP1-2	20	30	0	0.75
CAP1-3	60	30	40	0.75
CAP1-4	60	30	0	0.75
CAP2-1	20	30	40	0.75
CAP2-2	20	30	0	0.75
CAP2-3	60	30	40	0.75
CAP2-4	60	30	0	0.75
CAP3-1	20	30	40	0.75
CAP3-2	20	30	0	0.75
CAP3-3	60	30	40	0.75
CAP3-4	60	30	0	0.75

Supplementary Figure 5. Formulation table of CAP LNPs for *in vitro* luciferase assay.

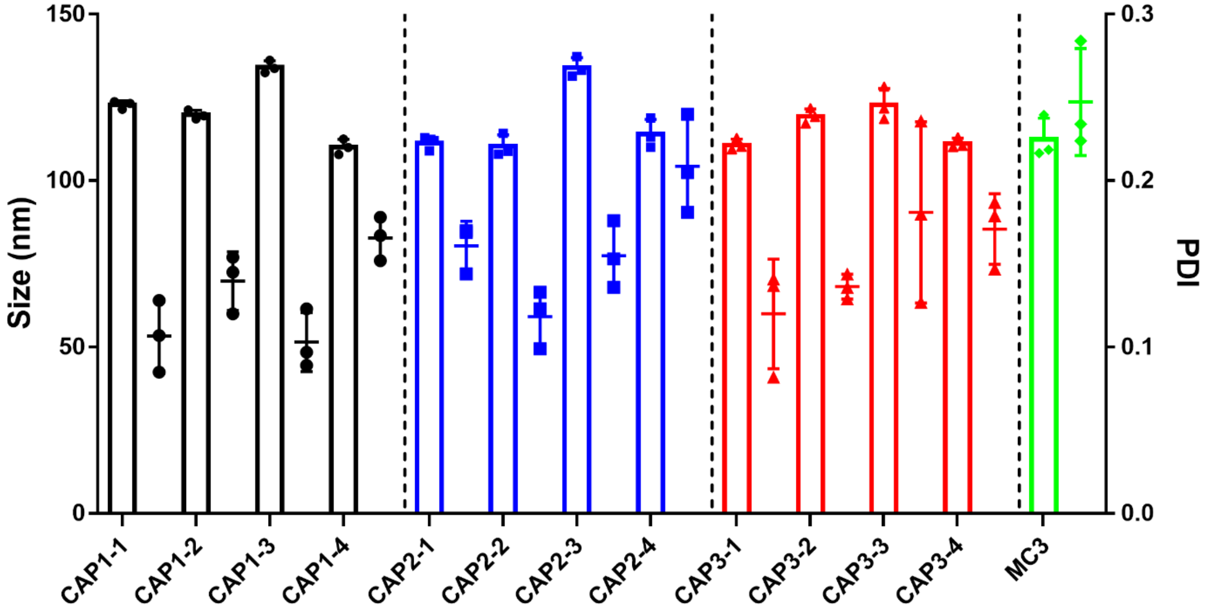

Supplementary Figure 6. Size and PDI of CAP-LNPs and MC3 LNPs measured by DLS.

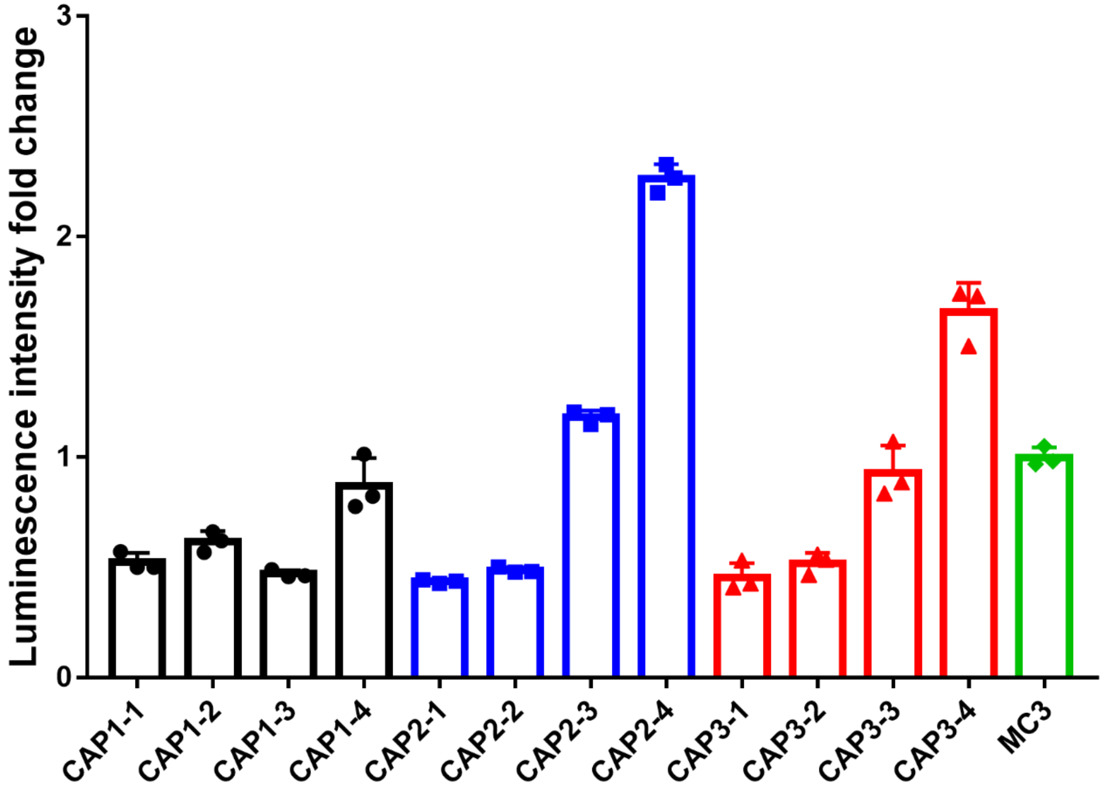

Supplementary Figure 7. *In vitro* delivery efficiency of FLuc mRNA encapsulated CAP-LNPs as compared to MC3 LNPs in Hep3B cells.

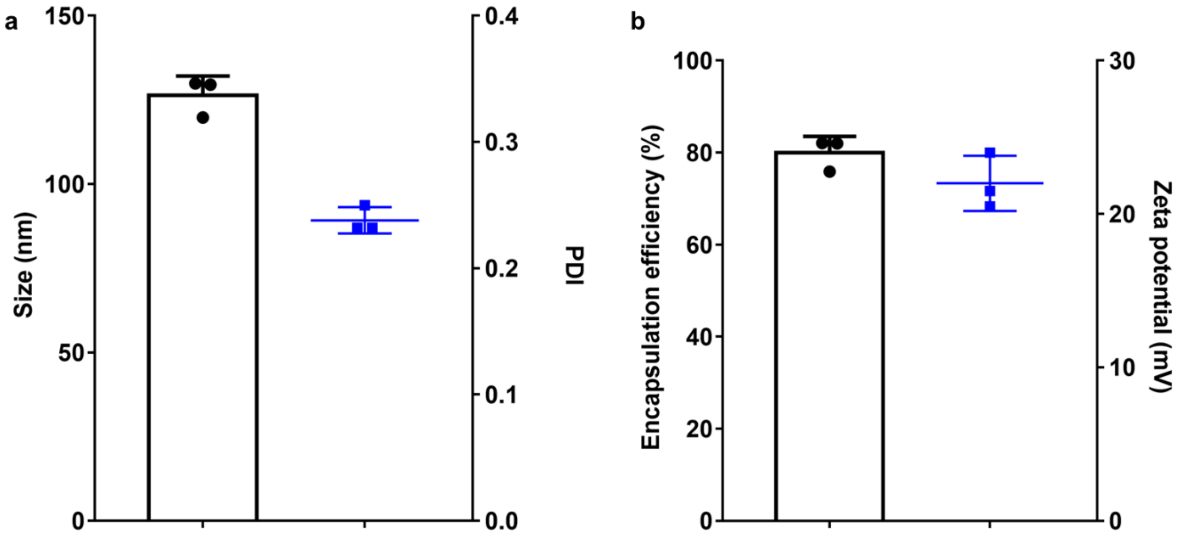

Supplementary Figure 8. a, Size and polydispersity index (PDI) of CAP2-4 LNPs measured by DLS. b, Encapsulation efficiency and zeta potential of CAP2-4 LNPs.

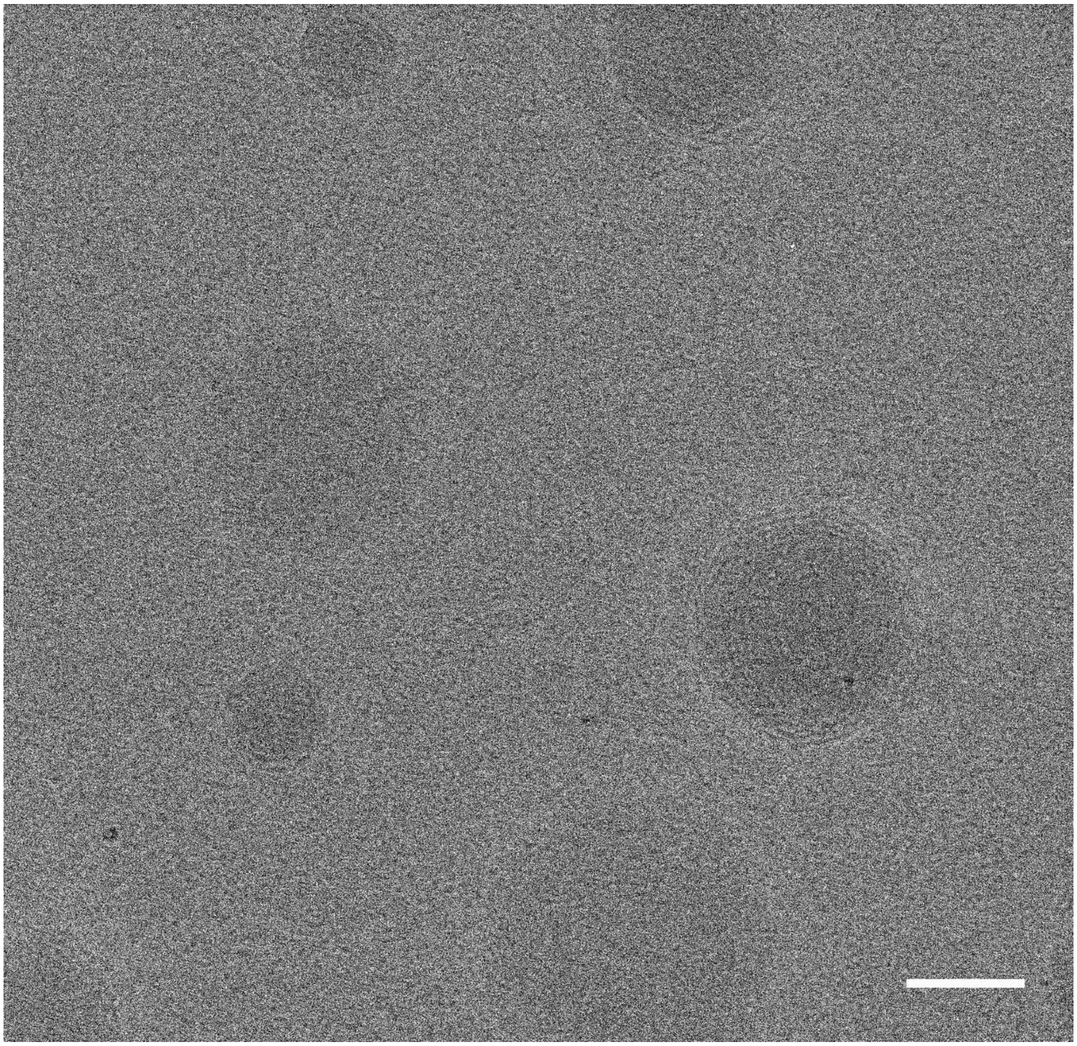

Supplementary Figure 9. Cryo-EM image of CAP2-4 LNPs including the representative nanoparticle shown in Figure 2d. Scale bar, 50 nm.

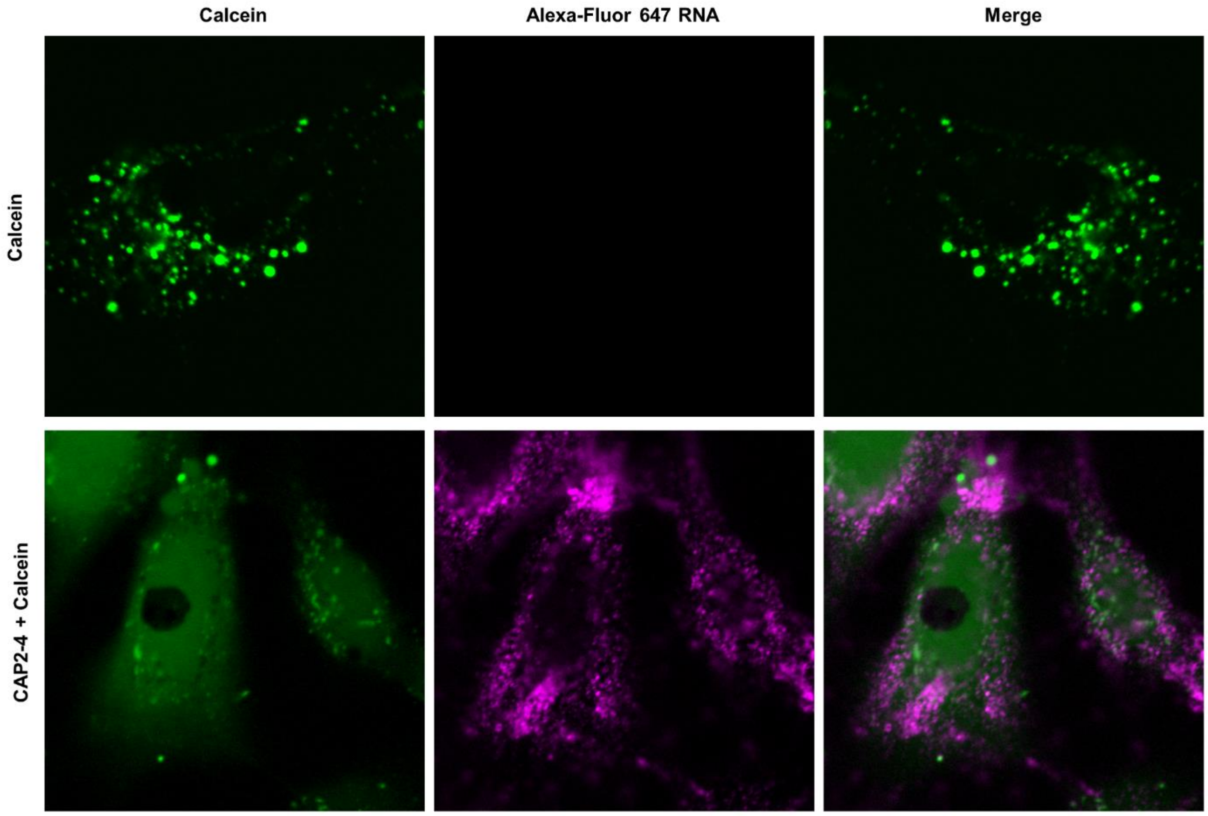

Supplementary Figure 10. Confocal images of Hep3B cells incubated with calcein alone or with CAP2-4 LNPs.

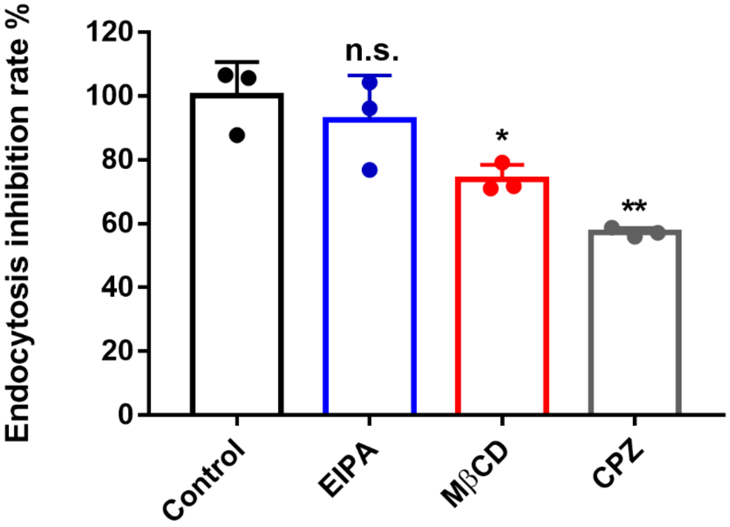

Supplementary Figure 11. Cellular uptake of CAP2-4 LNPs encapsulating Alexa-Fluor-647-labeled RNAs in the presence of endocytosis inhibitors, EIPA, MβCD and CPZ. Data are presented as the mean ± S.D. (n = 3). Statistical significance was analyzed by one-way ANOVA with Dunnett's multiple comparison test. *P < 0.05, **P < 0.01.

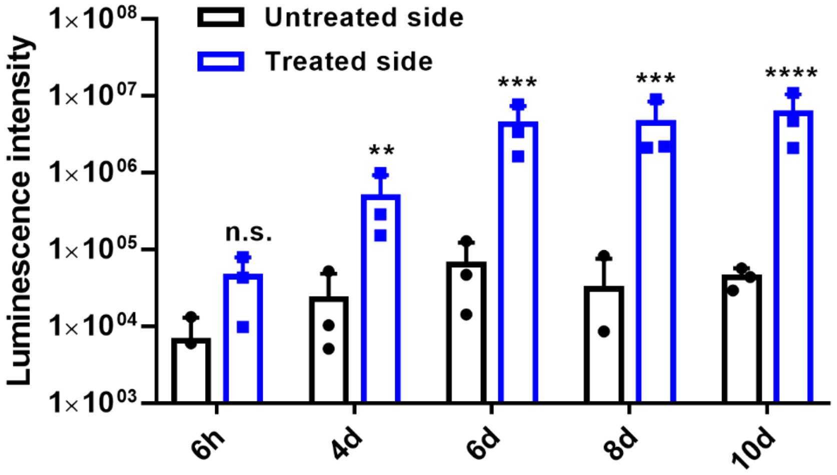

Supplementary Figure 12. Luciferase expression mediated by FLuc saRNA encapsulated CAP2-4 LNPs in the testes. saRNA-LNPs were microinjected into the left testis and the luciferase expression from the treated side (left testis) and untreated side (right testis) was monitored for 10 days. Data are presented as the mean ± S.D. (n = 3). Statistical significance was analyzed by the two-tailed Student's t-test. **P < 0.01, ***P < 0.001, ****P < 0.0001

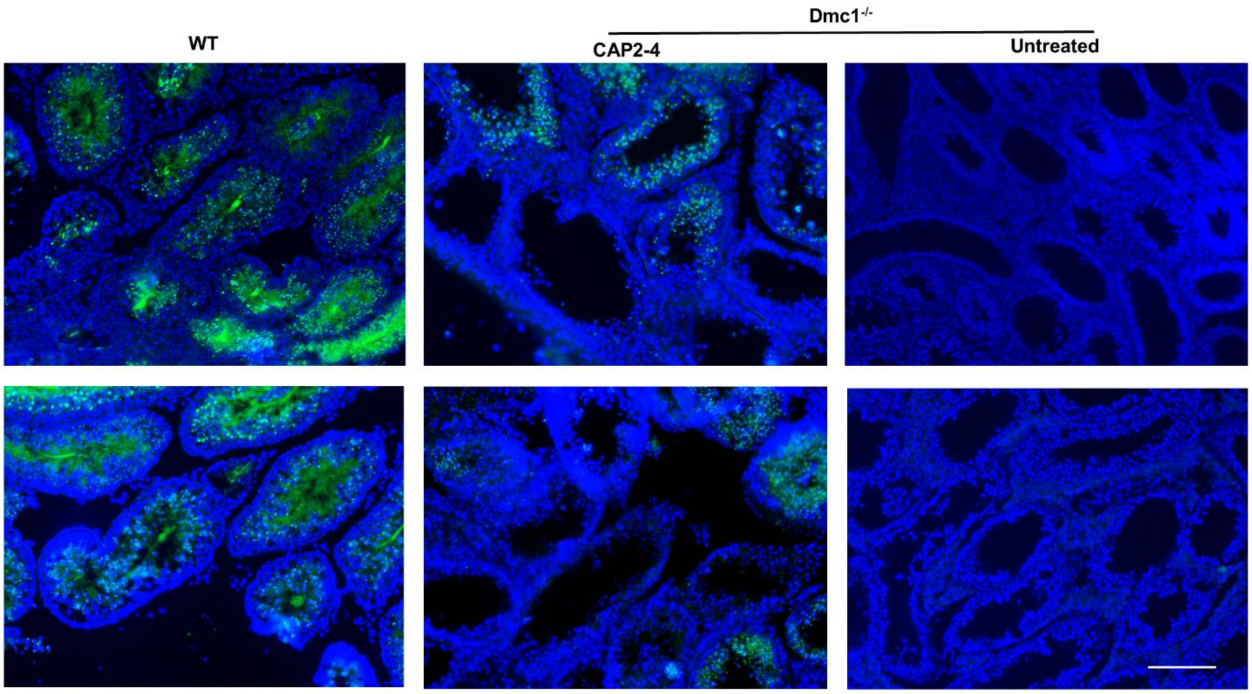

Supplementary Figure 13. Fluorescent images of PNA-lectin labeled spermatozoa of WT; Dmc1$^{-/-}$ mice treated with CAP2-4 LNPs, and untreated Dmc1$^{-/-}$ mice (testes). Scale bar: 50 µm.

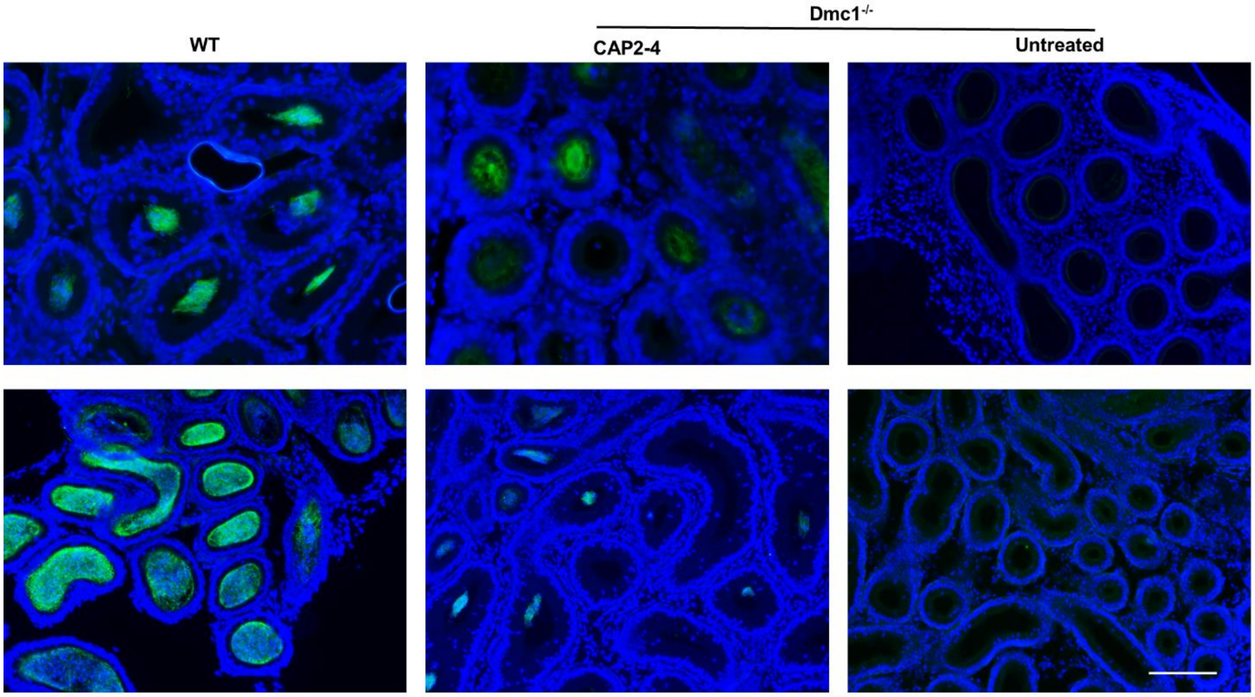

Supplementary Figure 14. Fluorescent images of PNA-lectin labeled spermatozoa of WT; Dmc1$^{-/-}$ mice treated with CAP2-4 LNPs, and untreated Dmc1$^{-/-}$ mice (epididymis). Scale bar: 50 µm.

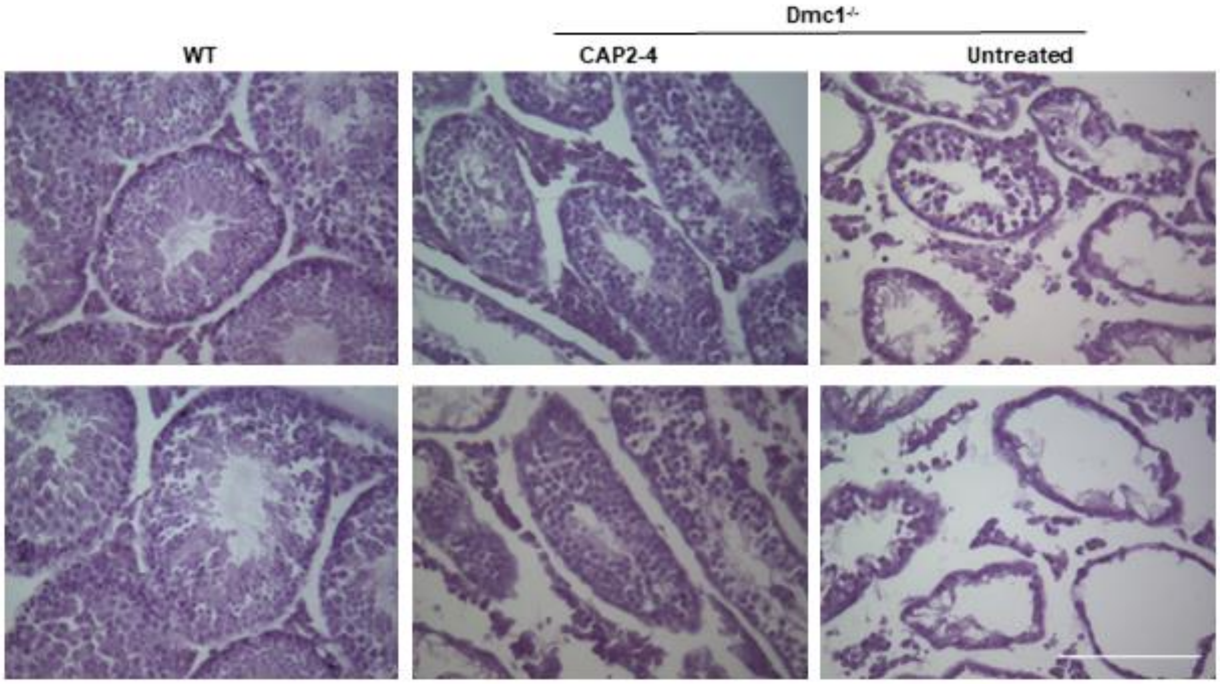

Supplementary Figure 15. Histological analysis of WT; Dmc1$^{-/-}$ mice treated with CAP2-4 LNPs, and untreated Dmc1$^{-/-}$ mice at 20× magnifications. Scale bar: 50 μm.

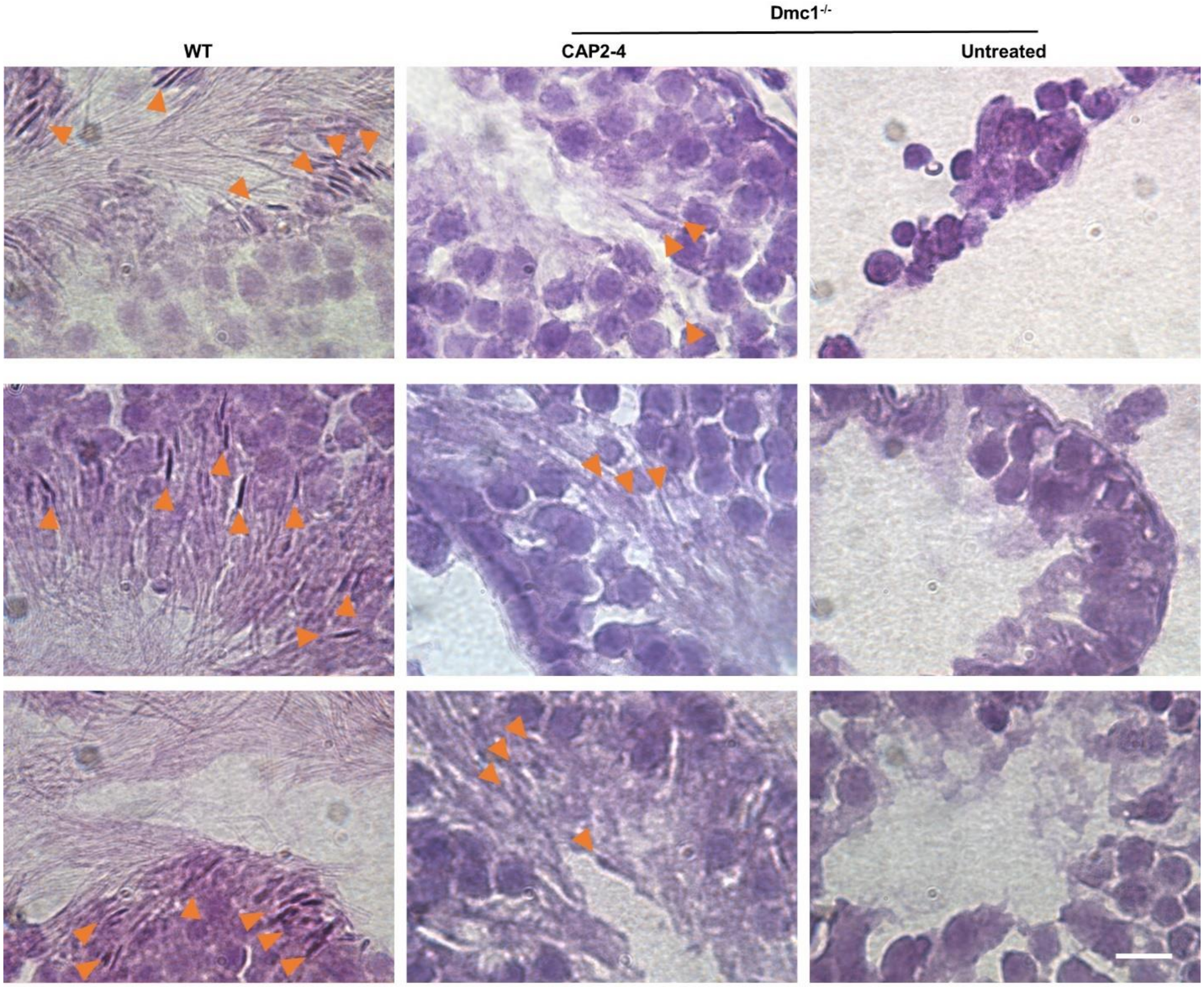

Supplementary Figure 16. Histological analysis of WT; Dmc1$^{-/-}$ mice treated with CAP2-4 LNPs, and untreated Dmc1$^{-/-}$ mice at 100× magnifications. The yellow arrows mark the mature spermatids. Scale bar: 5 µm.

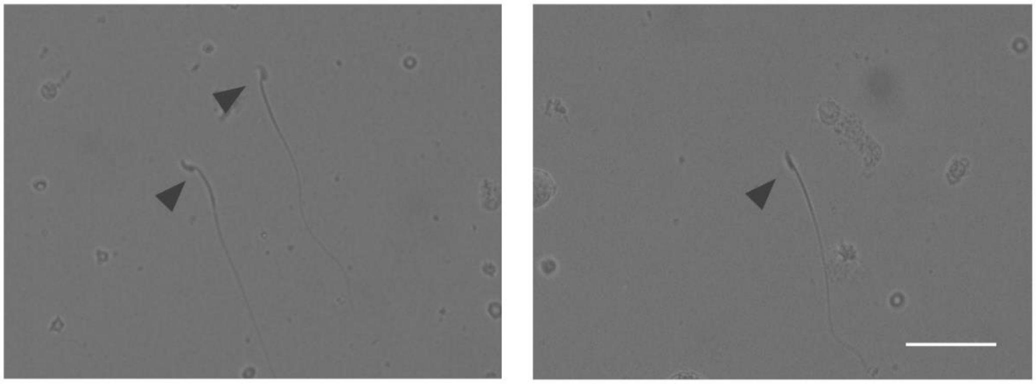

Supplementary Figure 17. Microscopy analysis of sperms from Dmc1$^{-/-}$ mice treated with CAP2-4 LNPs. The black arrows mark the sperms. Scale bar: 20 µm.
